# The Spa Physician

**Published:** 1902-05-10

**Authors:** 


### The Spa Physician.

A question has arisen lately as to the limitations which may properly be imposed upon the functions of the "spa physician." According to old ideas he was a practitioner who by dint of much experience of the baths or waters or forms of hydropathic treatment for which his particular spa was celebrated, was able to give useful assistance to the visitor, telling him in what quantity, at what times, and in what manner the healing waters had best be applied. In that way he fulfilled a useful although a somewhat humble mission. The patient never became "his case," but remained under the care of his own doctor, to whom, when he had been duly bathed and watered, he returned to report results, and who, be it added, took all responsibility, often being abused in no measured terms for having sent the patient to the wrong place if results did not come up to expectations. The spa physician was indeed nothing more than a sort of head bath-man, who would for a consideration see the patient safely through the course for which the particular spa was famous. And this arrangement had a certain utility. Every watering - place has, by dint of years of experience of its own resources, developed a line of treatment more or less peculiar to itself, and it is to undergo such treatment that we send our patients. Thus when we advise them to go to Bath or to Buxton, to Aix or to Wildbad, we expect them to receive the treatment for which the bath we prescribe has become famous—the treatment which depends upon its own peculiar resources, and not that which the "spa physician" may happen to choose out of the multitude of medicines open to us all. We do not send our patient to Dr. So-and-So because we want his advice upon the case, or because we care a rap what he thinks about it, but merely because we want him to pass the patient through the "cure." The watering-place doctor, according to this view, is the presiding deity over his own bath, and nothing more. The spa physician of to-day, however, is by no means contented with this *rôle*. Having got his patient, he wants to do his best for him in the time allowed, and he, perhaps, not unreasonably, asks why he should be limited to the trumpery baths and the old-fashioned waters amid which it is his fate to practise? Indeed it is highly probable that many of the fraternity look with considerable contempt on both baths and waters. These things are good enough as means of drawing patients, but in all that concerns actual treatment they pin their faith upon what are now called the adjuncts of spa treatment; upon heat and superheat; upon vapours and pulverisations; upon galvanism and sinusoidal currents; upon massage, "exercises," and vibrations—in fact, upon what the country doctor would, not entirely inaptly, describe as "all the quackeries." Referring to this subject at a recent meeting of the Balneological Society, Dr. Hedley said with much force that a health resort was a place endowed with special natural advantages which could not be acquired or imitated elsewhere and by which patients were attracted, and that if a patient were so attracted, say, to Harrogate, for the sake of its sulphur water or its moorland air, it might "savour somewhat of false pretence to immediately begin to roast him with luminous heat, or batter him with vibrations, or bathe his nude body in blue light, or bombard him with high-frequency currents." Yet the spa physicians have much to say in their own defence, and much that is unanswerable. On the one hand, they truly assert that, owing to the large number of cases of certain diseases which gravitate to certain spas, they may properly claim for themselves the *status* of specialists in regard to those ailments, while those who practise in places which are equipped with the elaborate apparatus required for the conduct of certain well-recognised modes of treatment—treatment which cannot be obtained elsewhere—may properly enough maintain that they would be wasting their opportunities and failing in their duty to their patients were they not to avail themselves of the advantages they enjoy.

The fact, we think, has to be recognised that in certain directions the treatment of disease by physical methods has become such an elaborate affair that the patient must needs go to the apparatus rather than the apparatus to the patient, and when this is the case it is obviously appropriate that such treatment should be carried out in health resorts which, from their very name, one may reasonably presume to be places conducive to the health of those residing in them. At the same time it is evident that some clear understanding should be arrived at when a patient is sent to a watering-place for the sake of undergoing a particular line of treatment. It would be absurd for a patient who had been sent to a place famed for its pure air, to find himself shut up in a box breathing stifling vapours and suffocative antiseptics, whatever the intrinsic advantages of such treatment might appear to the spa physician.

